# Supplementary material for: A TRIM insertion in the promoter of Ms2 causes male sterility in wheat
Source: Nat Commun. 2017 May 12;8:15407. doi: 10.1038/ncomms15407 (PMC5437302; doi:10.1038/ncomms15407)
Supplement: Supplementary Information — Supplementary Figures and Supplementary Tables [file ncomms15407-s1.pdf]

Supplementary Figures

Supplementary Fig. 1

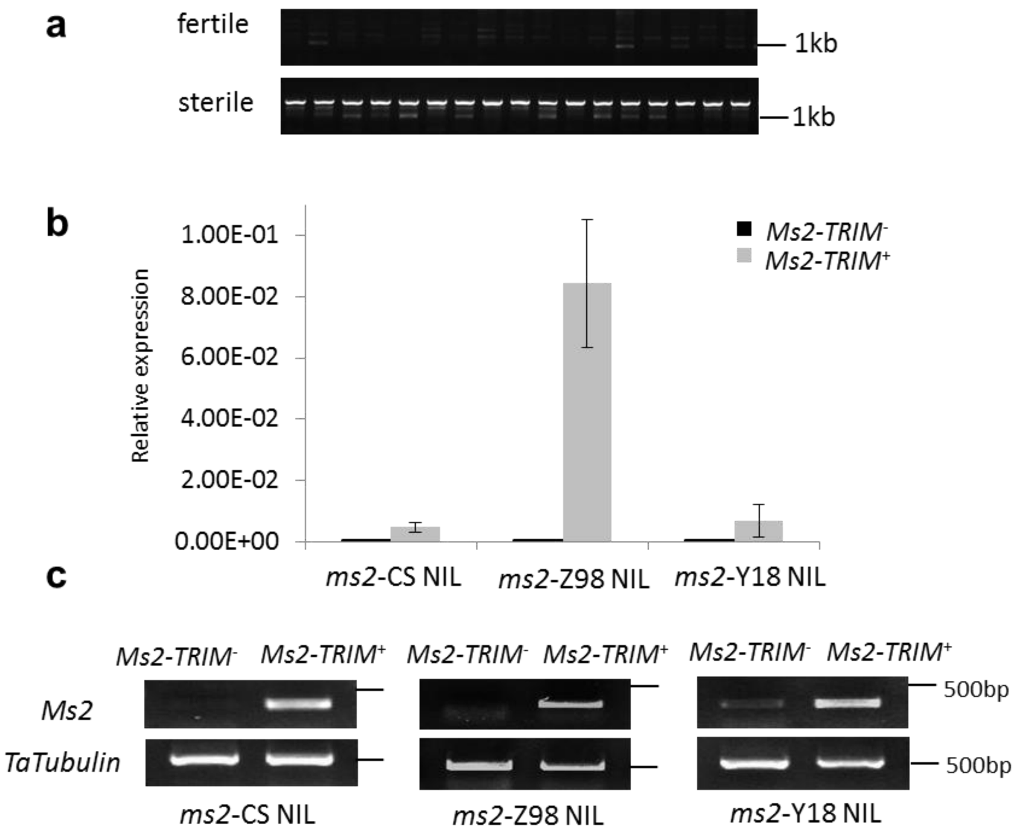

Supplementary Figure 1 ︱ *Ms2* polymorphism and expression pattern analysis. (**a**) TRIM insertion status in 17 different *ms2* NILs detected by a PCR marker amplifying a portion of the TRIM element sequence and *Ms2* promoter. (**b, c**) *Ms2* expression in the anthers of WT (*Ms2-TRIM*$^{-}$, fertile) and *ms2* mutant (*Ms2-TRIM*$^{+}$, sterile) NIL plants in three genetic backgrounds (Chinese Spring, Zhengmai98, and Yumai18).

Supplementary Fig. 2

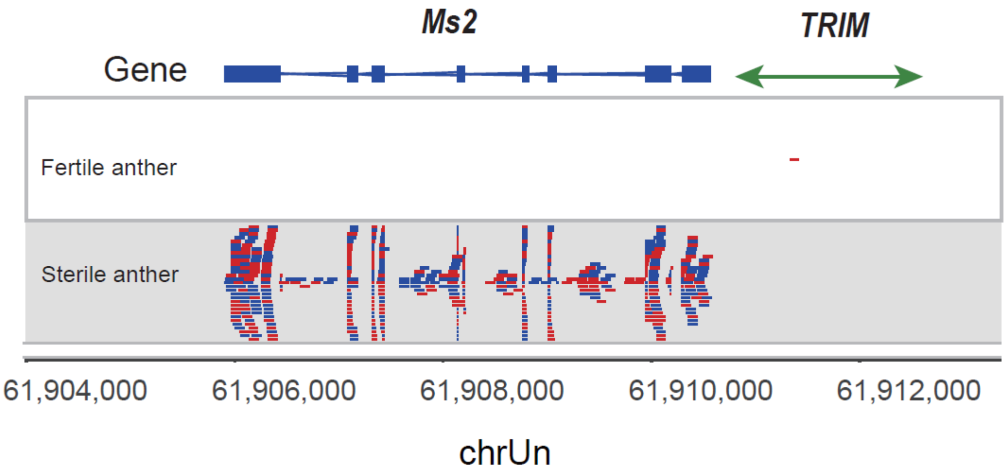

Supplementary Figure 2 | The reads distribution across the TRIM element and gene no.14. RNA-seq reads from the anther libraries from the ms2 mutant and wild type wheat were mapped to an artificial wheat genome (Chinese Spring) with the Ms2-TRIM sequence inserted in front of gene no.14. The plot is adopted from SeqMonk, and the red and blue blocks represent reads mapped to forward mapping and reverse strands, separately.

Supplementary Fig. 3

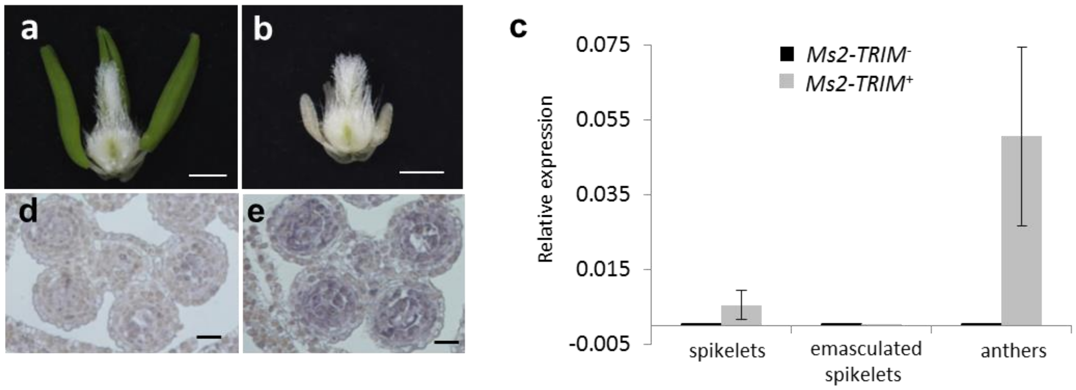

Supplementary Figure 3 ︱ The phenotype of Ai-Bai wheat and *Ms2* expression analysis. Phenotypes of floral organs, following the removal of the palea and lemma, of tall-fertile progeny of Ai-Bai wheat (*-/Ms2-TRIM*$^{-}$) (**a**) and dwarf-sterile progeny of Ai-Bai wheat (*Rht-D1c/Ms2-TRIM*$^{+}$) (**b**). (**c**) *Ms2* expression in three reproductive organs (spikelets, emasculated spikelets, anthers) of WT (*Ms2-TRIM*$^{-}$, fertile) and *ms2* mutant (*Ms2-TRIM*$^{+}$, sterile) plants in the Yanzhan1 genetic background. (**d, e**) *In situ* hybridization analysis (antisense probe) of *Ms2* in cross-sections of late prophase stage anthers from WT (**d**) and *ms2* mutant (**e**) plants.

Bars = 500 μm in (**a**) and (**b**), and 50 μm in (**d**) and (**e**).

Supplementary Fig. 4

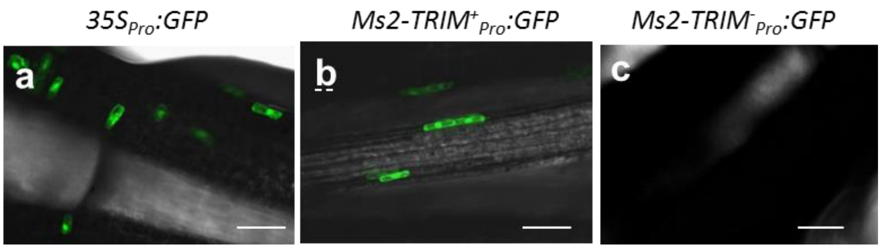

Supplementary Figure 4 │ Activity analysis of the *Ms2* promoter.

(**a-c**) GFP fluorescence in anther transiently expressing **$35S_{Pro}$:GFP** (**a**), **Ms2-TRIM$^{+}_{Pro}$:GFP** (**b**), and **Ms2-TRIM$^{-}_{Pro}$:GFP** (**c**).

Bars = 100 μm in (**a**) to (**c**).

Supplementary Fig. 5

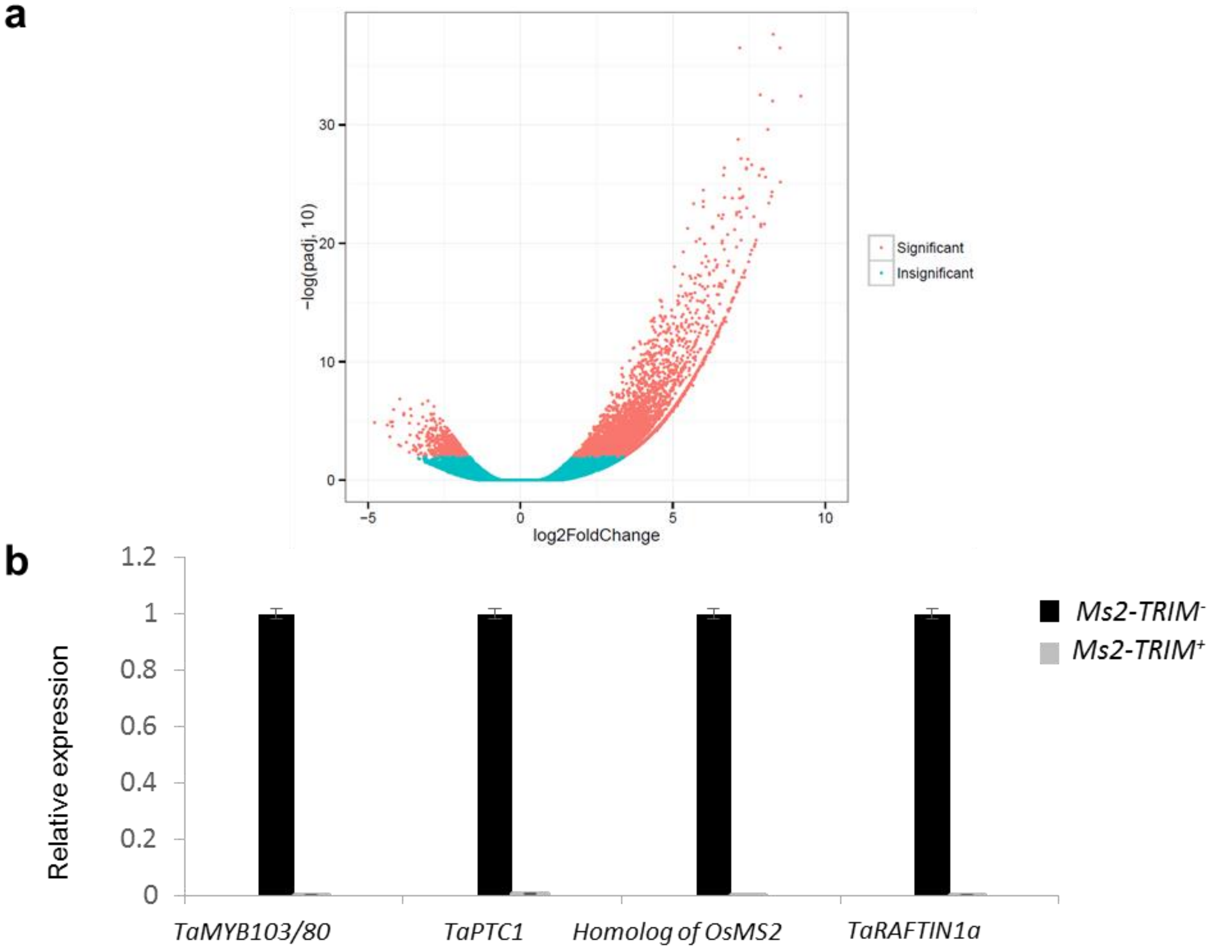

Supplementary Figure 5 │Differentially expressed genes between *ms2* mutant *vs.* WT. (**a**) Volcano plot of differentially expressed genes between *ms2* mutant *vs.* WT anthers. (**b**) The relative expression of *TaMYB80/MYB103*, *TaPTC1*, *Homolog of OsMS2* and *TaRAFTIN1* in WT and *ms2* mutant plants.

Supplementary Fig. 6

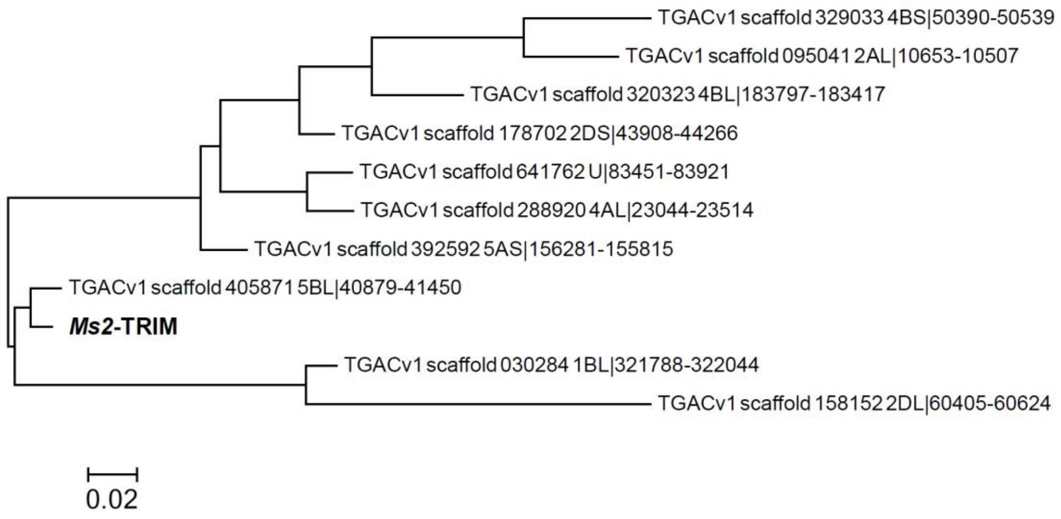

Supplementary Figure 6 │ Phylogenetic tree of *MS2-TRIM* and its hits in the hexaploid wheat genome (Chinese Spring).

Unrooted Neighbor-Joining phylogeny of the *Ms2* promoter TRIM based on the LTR region of the element in the wheat genome.

Supplementary Fig. 7

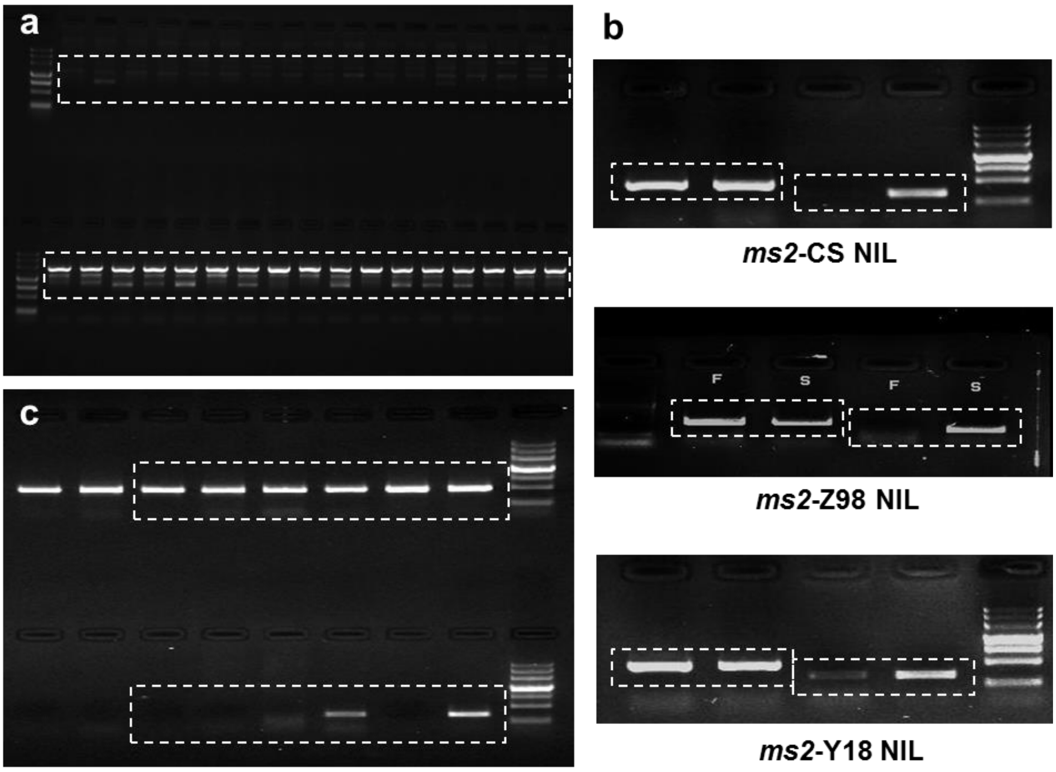

Supplementary Figure 7 | Uncropped gel images. (**a**) Uncropped gel image of supplementary figure 1a. (**b**) Uncropped gel images for RT-PCR in supplementary figure 1c. (**c**) Uncropped gel image for expression pattern analysis in figure 2a.

Supplementary Tables

Supplementary Table 1. Estimates of nucleotide diversity and test of statistics for selection in *Ms2*

	n	π(per site)	θ(per site)	Tajima's D	Fu and Li's D
Wild	30	0.00615	0.00585	0.176 $^{\$}$	0.381 $^{\$}$
Landrace	28	0.00386	0.00233	2.026*	0.704 $^{\$}$
Modern Variety	26	0.00302	0.00238	0.847 $^{\$}$	0.720 $^{\$}$

Note : π : nucleotide diversity , θ: Watterson’s estimator of genetic diversity, Statistical significance: $ P>0.1, * P <0.05

Supplementary Table 2. Materials were used to explore the nucleotide diversity of *Ms2*

**Accession/Sample names**	**Species**	**Abbr. Population Name**
TA1618	*Aegilops tauschii meyeri*	wild diploid D
TA2456	*Aegilops tauschii strangulata*	wild diploid D
TA2457	*Aegilops tauschii typica*	wild diploid D
TA2460	*Aegilops tauschii typica*	wild diploid D
TA2464	*Aegilops tauschii strangulata*	wild diploid D
TA2561	*Aegilops tauschii typica*	wild diploid D
TA1645	*Aegilops tauschii strangulata*	wild diploid D
TA1662	*Aegilops tauschii var.tauschii*	wild diploid D
TA2461	*Aegilops tauschii var.typica*	wild diploid D
TA2463	*Aegilops tauschii strangulata*	wild diploid D
TA2474	*Aegilops tauschii var.tauschii*	wild diploid D
TA2475	*Aegilops tauschii var.tauschii*	wild diploid D
TA2529	*Aegilops tauschii var.meyeri*	wild diploid D
CLAE1	*Aegilops tauschii*	wild diploid D
CLAE5	*Aegilops tauschii*	wild diploid D
CLAE17	*Aegilops tauschii*	wild diploid D
CLAE25	*Aegilops tauschii*	wild diploid D
PI 268210	*Aegilops tauschii*	wild diploid D
RL5214	*Aegilops tauschii*	wild diploid D
RL5257	*Aegilops tauschii*	wild diploid D
RL5261	*Aegilops tauschii*	wild diploid D
RL5263	*Aegilops tauschii*	wild diploid D
RL5272	*Aegilops tauschii*	wild diploid D
RL5392	*Aegilops tauschii*	wild diploid D
RL5422	*Aegilops tauschii*	wild diploid D
Y221	*Aegilops tauschii ssp.Stran gulata Eig.*	wild diploid D
Y222	*Aegilops tauschii ssp.strangulata EIG.*	wild diploid D
Y293	*Aegilops tauschii ssp.meyeri GRISEB.*	wild diploid D
Y294	*Aegilops tauschii ssp.meyeri GRISEB.*	wild diploid D
Y296	*Aegilops tauschii ssp.strangulata EIG.*	wild diploid D
sankecun	*Triticum asetivum*	landrace
zijuhong	*Triticum asetivum*	landrace
lanxizaoxiaomai	*Triticum asetivum*	landrace
heshangmai	*Triticum asetivum*	landrace
hongmangyouzimai	*Triticum asetivum*	landrace
tumangmai	*Triticum asetivum*	landrace
jiyumai	*Triticum asetivum*	landrace
changmangyingkemai	*Triticum asetivum*	landrace
nuomai	*Triticum asetivum*	landrace
wuxumai	*Triticum asetivum*	landrace
baihuamai	*Triticum asetivum*	landrace

dahongmai	*Triticum asetivum*	landrace
xinganxiaomai	*Triticum asetivum*	landrace
sanyuehuang	*Triticum asetivum*	landrace
dabaimai	*Triticum asetivum*	landrace
huangguaxian	*Triticum asetivum*	landrace
baiyoumai	*Triticum asetivum*	landrace
huajiechangbaican	*Triticum asetivum*	landrace
sanyuanmai	*Triticum asetivum*	landrace
shanmai	*Triticum asetivum*	landrace
sifangmai	*Triticum asetivum*	landrace
tuotuomai	*Triticum asetivum*	landrace
yangmai	*Triticum asetivum*	landrace
qigongmai	*Triticum asetivum*	landrace
chiyacao	*Triticum asetivum*	landrace
changgongfangxingmai	*Triticum asetivum*	landrace
yunnanxiaomai (YN3)	*Triticum asetivum*	landrace
paozimai	*Triticum asetivum*	landrace
bainong 791	*Triticum asetivum*	cultivated line
chuanmai 22	*Triticum asetivum*	cultivated line
chuanmai 42	*Triticum asetivum*	cultivated line
fengkang 7	*Triticum asetivum*	cultivated line
jinan 16	*Triticum asetivum*	cultivated line
jinan 4	*Triticum asetivum*	cultivated line
jinan 8	*Triticum asetivum*	cultivated line
jimai 23	*Triticum asetivum*	cultivated line
jingnong 79-13	*Triticum asetivum*	cultivated line
kefeng 6	*Triticum asetivum*	cultivated line
kehan 10	*Triticum asetivum*	cultivated line
liaochun 9	*Triticum asetivum*	cultivated line
longmai 33	*Triticum asetivum*	cultivated line
lumai 3	*Triticum asetivum*	cultivated line
mianyang 29	*Triticum asetivum*	cultivated line
neimai 19	*Triticum asetivum*	cultivated line
ningmai 13	*Triticum asetivum*	cultivated line
nongda 36	*Triticum asetivum*	cultivated line
shanhe 6	*Triticum asetivum*	cultivated line
shannong 1	*Triticum asetivum*	cultivated line
shijiazhuang 407	*Triticum asetivum*	cultivated line
wanmai 50	*Triticum asetivum*	cultivated line
xibei 612	*Triticum asetivum*	cultivated line
xiannong 39	*Triticum asetivum*	cultivated line
xiaoyan 4	*Triticum asetivum*	cultivated line
xiaoyan 759	*Triticum asetivum*	cultivated line

Supplementary Table 3. Primers used in map-based cloning and RT-qPCR

primer name	sequence 5' – 3'	type
M1-Forward	GGTATGCTCCTCATTTATTCC	SSR
M1-Reverse	GACTAGTTCATCTTGCATTCAC	
M2-Forward	TAGTATTTGAGAGAGAGGAGCTG	SSR
M2-Reverse	TTTTGTTCGTCCTGATCTGA	
M3-Forward	TCGGATCCTTCACTGATGTGCT	SNP
M3-Reverse	TCCTGGCAAGTCTGCATCTTCA	
M4-Forward	TGCACTTTAGAGCTCTGCACCA	SNP
M4-Reverse	GGTGAGTGTGTGTGTTTGTCAGCA	
M5-Forward	TTGGCTTGCCATTGCCATCGTC	SNP
M5-Reverse	CAAACTTTGCAGCCTCAACTTG	
M6-Forward	AGGGTGAGTAAGATCGTCAGCA	SNP
M6-Reverse	GAAGTACTGATTGATGTGAGGAC	
M7-Forward	GGCGTCCTCGTCTCCCAATA	Insertion
M7-Reverse	CGATGATGAACTCACCTGCTGTA	
M8-Forward	CCATACGAAAGCATAGCAATA	SSR
M8-Reverse	GTGATTGTGAGCAGTCCATT	
M9-Forward	CCACAAAATCAATGGTTTCTA	SSR
M9-Reverse	AACATTTTTAGACGTTTGACG	
M10-Forward	GGAGGCATAATGGAGAATG	SSR
M10-Reverse	CGTCTGTTTTCTCTCTCATCA	
M11-Forward	CTTACATGCAGATCCGTCTAAT	SSR
M11-Reverse	ATTTGTGTGTGTGTCTCTCTCT	
TaGAPDH-Forward	TTAGACTTGCGAAGCCAGCA	
TaGAPDH-Reverse	AAATGCCCTTGAGGTTTCCC	
TaTUBULIN-Forward	TGAGGACTGGTGCTTACCGC	
TaTUBULIN-Reverse	GCACCATCAAACCTCAGGGA	
Ms2-RT-Forward	CTGCTGCATCCGACTAACTATC	
Ms2-RT-Reverse	TGAGAATACTGTCCACCAAACTC	
